# Quantitative study for control of air–liquid segmented flow in a 3D-printed chip using a vacuum-driven system

**DOI:** 10.1038/s41598-022-13165-6

**Published:** 2022-05-28

**Authors:** Hyeonji Hong, Jae Min Song, Eunseop Yeom

**Affiliations:** 1 School of Mechanical Engineering, Pusan National University, Busan, South Korea; 2 Department of Oral and Maxillofacial Surgery, School of Dentistry, Pusan National University, Yangsan, South Korea

**Keywords:** Biomedical engineering, Mechanical engineering

## Abstract

The formation of droplets or bubbles in a microfluidic system is a significant topic requiring device miniaturization and a small volume of samples. Especially, a two-phase segmented flow can be applied to micro-mixing for chemical reactions and the treatment of heat and mass transfer. In this study, a flow of liquid slugs and bubbles was generated in a 3D-printed chip and controlled by a single pump creating a vacuum at the outlet. The pump and chip device were integrated to form a simple and portable system. The size and flow rate of liquid slugs, obtained through image processing techniques, were analyzed considering several parameters related to hydraulic resistance and pressure drop. In addition, the effect of segmentation on mixing was observed by measuring the intensity change using two different colored inks. The hydraulic resistance of air and liquid flows can be controlled by changing the tube length of air flow and the viscosity of liquid flow. Because the total pressure drop along the channel was produced using a single pump at the outlet of the channel, the size and flow rate of the liquid slugs showed a near linear relation depending on the hydraulic resistances. In contrast, as the total pressure varied with the flow rate of the pump, the size of the liquid slugs showed a nonlinear trend. This indicates that the frequency of the liquid slug formation induced by the squeezed bubble may be affected by several forces during the development of the liquid slugs and bubbles. In addition, each volume of liquid slug segmented by the air is within the range of 10^–1^ to 2 µL for this microfluidic system. The segmentation contributes to mixing efficiency based on the increased homogeneity factor of liquid. This study provides a new insight to better understand the liquid slug or droplet formation and predict the segmented flow based on the relationship between the resistance, flow rate, and pressure drop.

## Introduction

The generation of droplets or bubbles in the microfluidic system has been investigated in various fields such as medical, biological, and chemical research^1–6^. Two immiscible fluids can be utilized for micro-mixing^7^, implying a segmented flow due to the mutual relation between them in a micro-device. The introduced phases can generate the segmented flow including droplets, plugs, or slugs, and it is generally regarded as droplet formation^8^. This is considered as a significant topic in microfluidics because the development of point-of-care test (POCT) needs miniaturization of the system and small volume of the test sample. Therefore, predicting and controlling the formation of droplets or bubbles is important for various applications^9–12^.

In particular, the gas–liquid flow enables fast mixing and tolerates high temperature during chemical reactions^3^. Mixing performance in liquid would increase with short slug length and high velocity while it would also be constrained by short residence time. On the other hand, flow is not completely developed as the size of liquid slug is too short^13^. Therefore, it is important to controlling the size of slug (or droplet) and the ratio of gas-liquid flow rate. Besides the feature of gas–liquid flow, droplet formation process also develops mixing by the molecular diffusion due to squeezed and stretched interface^14^. The formation of segmented flow composed of gas–liquid has been studied through the experiments and simulations^1,15–25^. Visualization such as micro-PIV experiment was conducted for observing the velocity distribution including the recirculation motion in the liquid slug^1,23^. The researches on gas–liquid segmented flow have investigated the increased heat and mass transfer in liquid slug^25^, the size of liquid slug^16,17^, the influence of liquid properties on the flow^17,25^, the relation between pressure and flow^18–22^, the flow characteristics with capillary number^17,19^ using various designs of the channel such as T-junction.

In most studies, gas and liquid flows are injected into each inlet by each of two pumps because it is convenient to control the ratio between gas and liquid separately. For example, gas pressure is used to provide automatic generation of sequential flow, known as a self-activated flow^26,27^. Wu et al. proposed a pressure driven method of injecting a sample using a hand-held syringe into a chip^28^. However, in this method, each sample should be loaded into the supply chambers, which then flows through the connecting line. It means that much of the sample is consumed in the connecting line without mixing or reaction. Therefore, besides controlling the microscale segmented flow, the vacuum-driven flow by negative pressure using a single pump would be appropriate to achieve the smaller volume of the sample for devices or systems such as POCT. A few studies conducted experiments for segmented flow by negative pressure and most of them regulate liquid–liquid flows^29,30^. Garstecki et al. has reported that the movement of gas–liquid was operated by negative pressure for the simple and portable systems which is the integration of operating equipment and microfluidic devices^15^.

In case of a microfluidic-based device, repetitive modification is essential for a successive cycle of design, fabrication, and testing in the experimental stage. In addition, the ability to control the small volume of fluid is a typical need of the microfluidic system^31^. Due to this complex connection, it is difficult to apply the microfluidic-based devices to various industrial fields for widespread adoption and commercialization^32^. Hence, many challenges exist for POCT devices from laboratory to industrial application. To overcome these challenges, 3D (three-dimensional) printing technology, which is expected to provide novel methods in microfluidics, has been considered. It has advantages of easy and iterative design, and rapid fabricating of prototypes^33^. In addition, material can be reproduced at relatively low cost, and low volume production can be achieved with freeform design during trial and error^34^. Several types of 3D printing methods shows applicability and flexibility in view of microfluidic devices^35,36^. Many studies have reported the application to microfluidics^37–39^ and furthermore utility for the fluid separation or manipulation^33,34,40,41^ and diagnosis using biomarkers^40,42^. Bhargava et al. used 3D printing technology for fabricating a channel which can generate the liquid–liquid droplet^37^. It implies the possibility of optical measurement through selecting the appropriate printing materials.

In this study, negative pressure was applied to a microchip using a single-pump system to control the air–liquid flow. The micro-scale chip was fabricated using the 3D printing method for iterative modification to optimize design. The size and flow rate of the segmented flow were analyzed depending on several parameters that influence the hydraulic resistance of the fluids and the total pressure drop in the 3D-printed chip. Different tendency of generated liquid slug and air bubble was investigated between changing resistances of air and liquid parts each, and effect of pressure drop was observed by regulating the flow rate for vacuum-driven flow. Furthermore, the intensity change was measured for ascertaining mixing during formation of segmented flow by using two different colored fluids.

## Materials and methods

### Experimental setup

**Figure 1:**
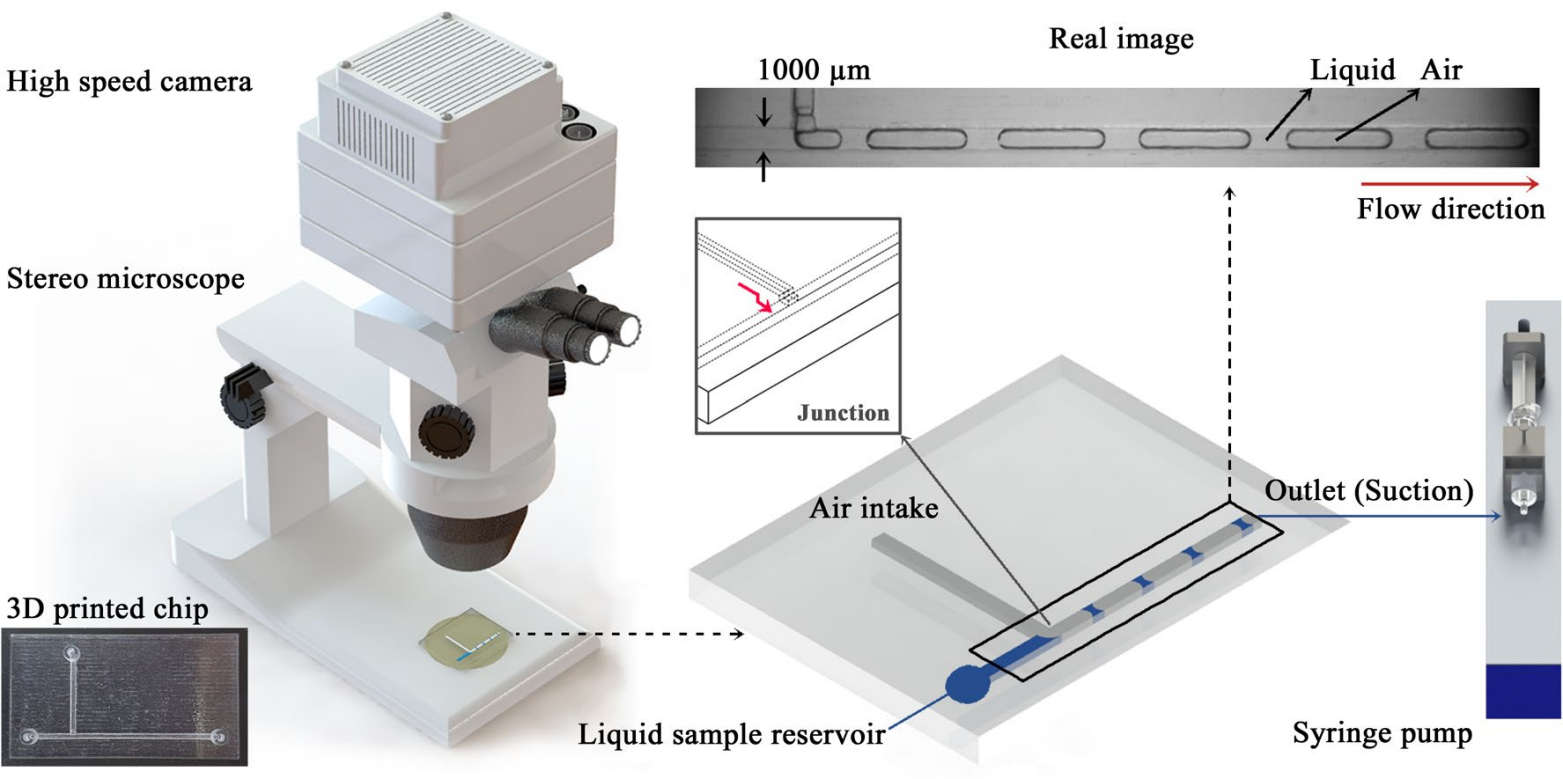
Experimental setup composed of a high-speed camera, a stereo microscope, a 3D-printed chip, and a syringe pump. T-shaped rectangular channel with a width and depth of 1000 µm for a mainstream channel with a length of 45 mm, and a depth of 500 µm for a 90° channel with a length of 20 mm. 3D illustrations were produced by the authors using SolidWorks software (Dassault Systèmes SolidWorks Corp., USA).

A 3D-printed chip was mounted on a stereo microscope (SZ61TR, Olympus, Tokyo, Japan) with light illumination and an objective lens at 1X magnification (numerical aperture (NA) = 0.071), as shown in Fig. 1. Flow in the 3D-printed chip was captured using a high-speed camera (Phantom VEO710L, Vision Research Inc., Wayne, NJ, USA) at 100 fps through the microscope. Liquid sample (50–700 µL with flow rate condition) was filled in the reservoir of the 3D-printed chip, and the air entered through the air intake part of the chip. Vacuum-driven flow was induced using a syringe pump (neMESYS, Centoni Gmbh, Germany) with a plastic syringe of 10 mL (BD; Becton Dickinson, Franklin Lakes, USA) connected at the outlet of the chip. The pump, controlled by the program of computer, precisely produced the volume change of syringe. In this study, experiments were conducted in a thermo-hygrostat room (SKS-ACUD-05, Deahan Cleantech, South Korea) with a temperature of 20 °C and 55% relative humidity.

### 3D printing

A 3D model of microfluidic device was designed using SolidWorks (Dassault Systèmes SolidWorks Corp., USA) and converted into the STL format file for fabrication. The microchip was printed using a 3D printer (J850, Stratasys, Eden Prairie/Minneapolis, USA) based on the polyjet method, which jet drops of liquid photopolymer onto a build tray, and then solidifies by exposure to the UV light while the layers accumulate. The effect of surface roughness on the flow is reduced since boundary layer is thicker than absolute roughness and viscous sublayer adheres and flows along the surface for laminar flow condition^43^. It has a T-shaped rectangular channel that is 1000 µm in width and depth for a mainstream channel with a length of 45 mm. To ensure sufficient hydraulic resistance for the inflow of air, the depth is 500 µm for a 90° channel with a length of 20 mm. The inlet and outlet have a diameter of 1.5 mm to connect to a flowing tube (inner diameter = 0.5 mm, outer diameter = 1.5 mm; Tygon tube). As illustrated in Fig. 1, the T-junction channel is mostly used for the formation of droplets or bubbles^16–18,21,22,24,44^. In the 3D-printed chip, the air enters through the 90° channel and joins the liquid flow in the mainstream channel. After reaching the junction, the air and liquid form a segmented flow (bubbles and liquid slugs). As the inset of junction in Fig. 1, direction at the junction turns twice for preventing liquid from directly entering the air intake part. The 3D structure was made by a 3D printer as one-off object. The dimensions of channel were determined through an iterative process of design, fabrication, and test to form segmented flows of gas–liquid. For example, the outlet of channel was redirected to be parallel with flow due to an obstacle of hydraulic pressure to the flow. As utilizing a 3D printing method, easy and freeform design, and rapid fabricating of prototype could be achieved with relatively low cost and low volume production during trial and error. Moreover, it would be used to extend the succeeding studies.

### Working fluids

The surface tension has an effect on the liquid–gas segmented flow^1,19^. For reducing the contribution of the interfacial force, low surface tension between ethanol-air was considered. Therefore, ethanol was used as a main working fluid and a mixture of ethanol–water was utilized for controlling the resistance of liquid sample. The viscosity and surface tension of the mixture of ethanol (1.1890 cP, 22.85 mN/m at 20 °C) and water (1.0030 cP, 72.88 mN/m at 20 °C) varied depending on the ethanol concentration. This viscosity variance was measured using a microfluidic viscometer based on pressure estimation developed in our previous research^34^. Ethanol concentration indicates volume percent, and the range of measurements is 0, 20, 40, 60, 80 and 100%. At 60% of ethanol concentration, viscosity has the maximum value of about 2.8990 cP. With reference to the value at 60% of concentration, viscosity showed a descending trend for both increase and decrease in ethanol concentration. The surface tension was obtained by interpolation technique based on the references on ethanol–water mixture^45,46^. In this study, pressure drop in the channel is within 10 Pa. Therefore, the compressibility of air is not considerable as the pressure is low and gas–liquid flow represents uniform patterns in the channel^11^. In addition, the solubility of air is very low under the low-pressure condition^47,48^. Therefore, air was used for two-phase segmented flow as a gas fluid^11,15,17,19,21,23^.

For visualizing the mixing by segmentation, two pigment inks (STORiA, SAILOR, Japan) were utilized for optical measurement. Each color is yellow and blue, and it was injected into the 3D-printed channel at 0.02 mL/min, respectively. After stabilization, mixing flow image was captured by smartphone camera (Galaxy S9 + edge, Samsung, South Korea) which was connected to the microscope lens using a smartphone camera adapter.

## Formation of liquid slug and bubble

**Figure 2:**
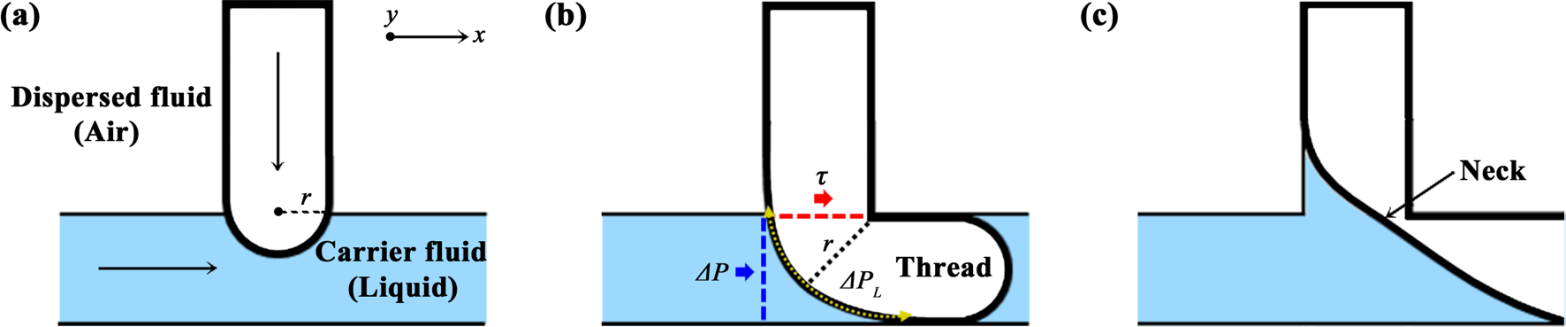
Process of developing segmented flow made up of liquid slugs and air bubbles in T-junction of 3D-printed chip. In this study, liquid in the main channel is considered a carrier fluid and air a dispersed fluid to be squeezed as flowing from 90° channel to main channel. Laplace pressure (Δ*P*_L_), shear stress (τ) and pressure drop (Δ*P*) are expressed as yellow dotted, red and blue arrows. The formation of the liquid slug and air bubble was illustrated by the authors using Adobe Photoshop 2021 (Adobe Inc., USA).

**Figure 3:**
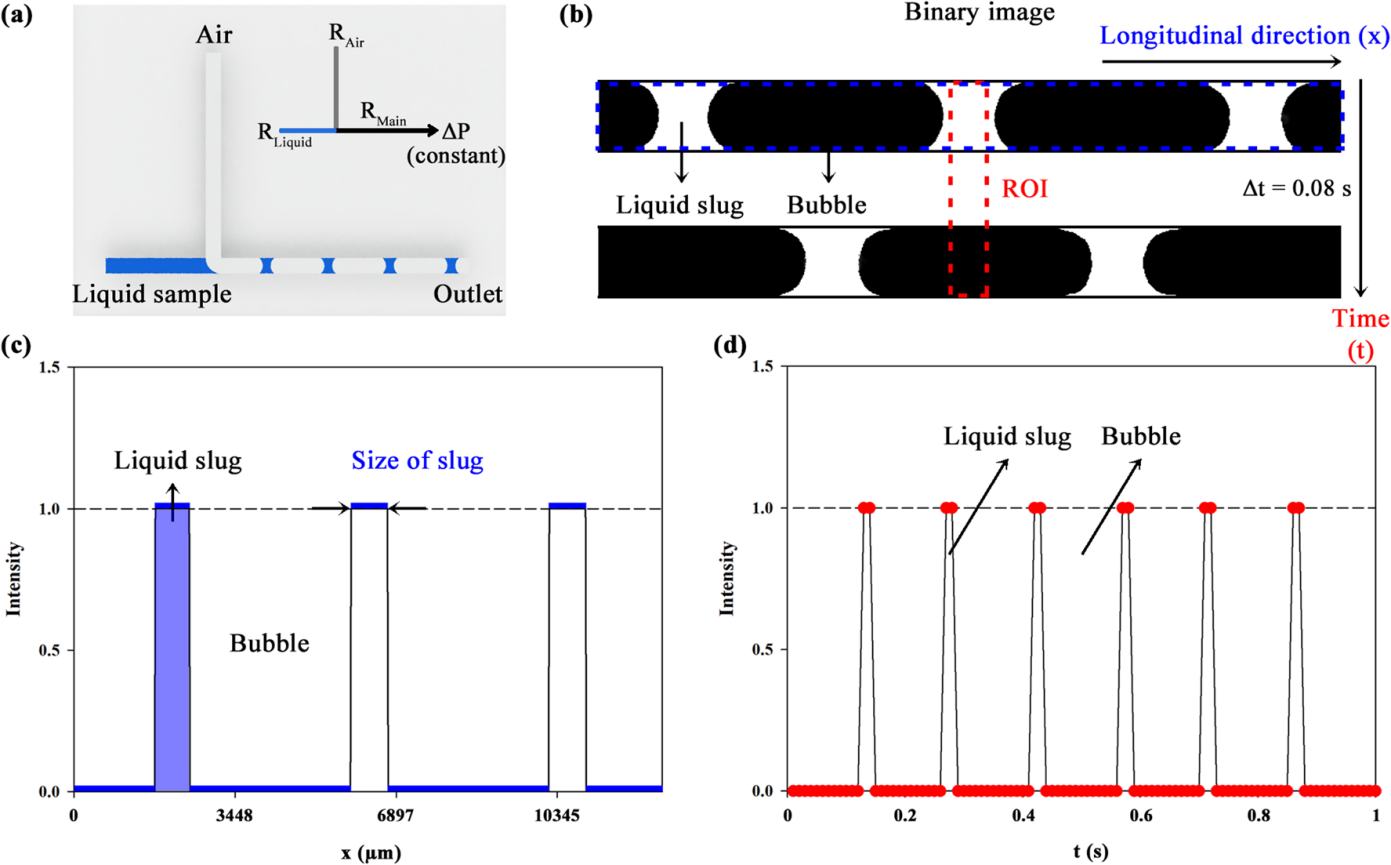
(**A**) Schematic describing hydraulic resistances of air (*R*_Air_) and liquid (*R*_Liquid_) flows analogous to the Ohm’s law. A 3D chip was illustrated by the authors using SolidWorks software. (**B**) Binary images converted using the Otsu thresholding method. In the obtained images (Δ*t* = 0.08 s), the white region represents liquid slugs (intensity value of 1) and the black region represents air bubbles (intensity value of 0). (**C**) Transverse intensity value depending on the longitudinal direction [*x* (µm)] at a certain time corresponding to blue box in (**B**). The size of liquid segment (*L*_S_) is shown in this graph. (**D**) Temporal variation of averaged intensity belonging to ROI depending on time (s) corresponding to red box in (**B**). The flow rate of liquid segment (*Q*_Liquid_) is shown in this graph.

In Fig. 1, both liquid and air flows in the channel are produced by the vacuum-driven flow using the syringe pump connected to the outlet of the microchip. The real image in the 3D-printed chip represents the segmented flow, including liquid and air. Figure 2 illustrates the formation of the liquid slug (carrier fluid) and air bubble (dispersed fluid). Among two-phase flow patterns, slug flow form shows the dispersion of gas bubbles and concave volume of liquid slugs under wide operating conditions^20^. For steady pressure-driven flow, the relation between flow rate and pressure drop is based on Hagen-Poiseuille’s law^16,49,50^. The constant pressure drop (*ΔP*) induced by one-pump system at the outlet can be expressed as follows:

1 $$\Delta P= {R}_{h}Q$$

where *R*_*h*_ is the hydraulic resistance and *Q* is the flow rate of the channel. In Fig. 3a, the hydraulic resistances of air and liquid flows, *R*_Air_ and *R*_Liquid_ respectively, are described analogous to the Ohm’s law. Simple resistance circuit model is physically reasonable in the air–liquid segmented flow^51^. Pressure term can be expressed for each section and rewritten. From that, pressure drop is obtained as follows:

2 $$\Delta {P}_{Air}= {R}_{Air}{Q}_{Air}+ {R}_{Main}({Q}_{Air}+ {Q}_{Liquid})$$

3 $$\Delta {P}_{Liquid}= {R}_{Liquid}{Q}_{Liquid}+ {R}_{Main}\left({Q}_{Air}+ {Q}_{Liquid}\right)$$

For Eqs. (2) and (3), two phases have a common term (*R*_Main_*(Q*_Air_ + *Q*_Liquid_)). In the above-mentioned experimental condition, *∆P*_Air_ and *∆P*_Liquid_ are the same as the syringe pump connected to the outlet controls the total pressure of the channel. As a result, the simplified Eq. (1) can describe the experimental results for each phase of air and liquid. Therefore, the hydraulic resistances of both air and liquid change the size and squeezing rate for air bubble and liquid slug because the hydraulic resistances vary the flow rate ratio between air and liquid under balanced forces (interfacial tension, shear stress, and hydrostatic pressure).

The obtained images (Δ*t* = 0.08 s) are converted into binary images using the Otsu thresholding method (Fig. 3b). The white region indicates the liquid slug with the intensity value of 1. On the contrary, the black region indicates the bubbles with the intensity value of 0. Figure 3c represents the transverse intensity at a certain time corresponding to the blue box including *x* (µm), which is the longitudinal direction in Fig. 3b. It was obtained by taking an average of intensity values along the channel width. The liquid slugs can be easily distinguished by high intensity, and thus, the size of the liquid segment (*L*_S_) can be acquired. Figure 3d illustrates the temporal variation of averaged intensity belonging to the region of interest (ROI). The ROI in Fig. 3b is considered smaller than the size of the liquid segment so that the liquid with the intensity value of 1 can be easily distinguished from the region occupied by the air. Using this, the number of liquid slugs during a certain period is estimated, and then the liquid proportion in the total flow is calculated. Consequently, the flow rate of liquid slugs (*Q*_Liquid_) is determined using the known total flow rate.

## Results

### Air resistance with length of tube

**Figure 4:**
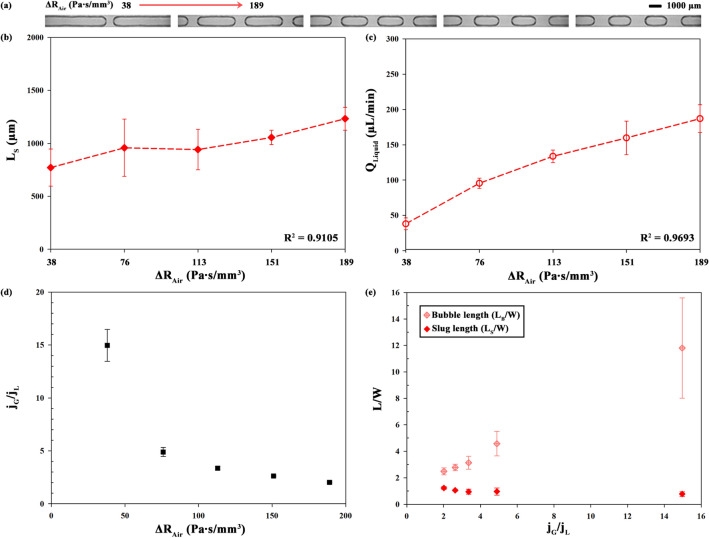
(**a**) Representative images in 3D-printed chip depending on hydraulic resistance gradient of air [Δ*R*_Air_ (Pa∙s/mm^3^)]. (**b**) Length (*L*_S_) of slug, (**c**) flow rate (*Q*_Liquid_) of slug and (**d**) air–liquid superficial velocity ratio (*j*_G_/*j*_L_) depending on Δ*R*_Air_. Correlation coefficients for the given length of tube are *R*^2^ = 0.9105(*L*_S_) and *R*^2^ = 0.9693 (*Q*_Liquid_). (**e**) Dimensionless length (*L*_S_/W, *L*_B_/W) versus air–liquid superficial velocity ratio (*j*_G_/*j*_L_).

To control the hydrodynamic resistance of the air part (*R*_Air_), an additional tube (D = 250 µm; Tygon tube) was connected to the air intake. Then, the length of the connected tube which is circular channel was controlled to change *R*_Air_. According to Eq.  (4), the *R*_Air_ increases as the tube lengthens.

4 $${R}_{h}= {C}_{geometry}\mu L/{A}^{2}= 8\mu L/\pi {a}^{4}$$

where *C*_geometry_ is a geometric coefficient, *µ* is the dynamic viscosity, *L* is the length, and *A* is the cross-sectional area of the channel. For a circular channel, *C*_geometry_ is 8π, and *a* is the radius of the channel^49^. The flow rate (*Q*_Pump_) was fixed at 500 µL/min, whereas the length of the tube was varied as 200, 400, 600, 800, and 1000 mm. From Eq. (4), *R*_Air_ changes from 38 to 189 Pa∙s/mm^3^ at the air viscosity of 1.81 × 10^–5^ Pa∙s according to the relevant length of tube. The increase in *R*_Air_ (Δ*R*_Air_) results in the decrease in the flow rate of the air (*Q*_Air_) under constant Δ*P* based on Eq. (1). Therefore, air flows in the main channel with a relatively low volume compared to the liquid part. Figure 4a–c show the length (*L*_S_) and flow rate (*Q*_Liquid_) of slugs depending on the length of tube that was represented by Δ*R*_Air_ (Pa∙s/mm^3^). The value of *L*_S_ increases gradually from 771 to 1232 µm (*R*^2^ = 0.9105). Likewise, the value of *Q*_Liquid_ increases from 38 to 187 µL/min with the length of tube.

In the case of *Q*_Liquid_, the correlation coefficient (*R*^2^ = 0.9693) is higher than that in the case of *L*_S_ (R^2^ = 0.9105) because *Q*_Liquid_ is directly affected by *Q*_Air_ (Δ*P* = *R*_Air_*Q*_Air_). For detailed explanation, Fig. 4d shows the air–liquid superficial velocity ratio (*j*_G_/*j*_L_) for the Δ*R*_Air_. Superficial velocity can be calculated by flow rate divided by cross-sectional area (A) (i.e. *j*_G_ = *Q*_Air_/A). When Δ*R*_Air_ is increased, *j*_G_/*j*_L_ is decreased. Figure 4e shows the considerable linear increase of dimensionless length of bubble (*L*_B_/W) with increase of (*j*_G_/*j*_L_) while length of slug (*L*_S_/W) is decreased (*L*_S_ = 0.77–1.23 µm). The trend of relation between *L*/W and *j*_G_/*j*_L_ is matched with gas–liquid microchannel system^20^. Therefore, Δ*R*_Air_ make the *j*_G_/*j*_L_ reduced and *L*/W is affected by *j*_G_/*j*_L_ under given liquid flow rate. Reduced gas flow rate results in shorter gas bubbles and higher liquid slugs.

### Liquid resistance with ethanol concentration

**Figure 5:**
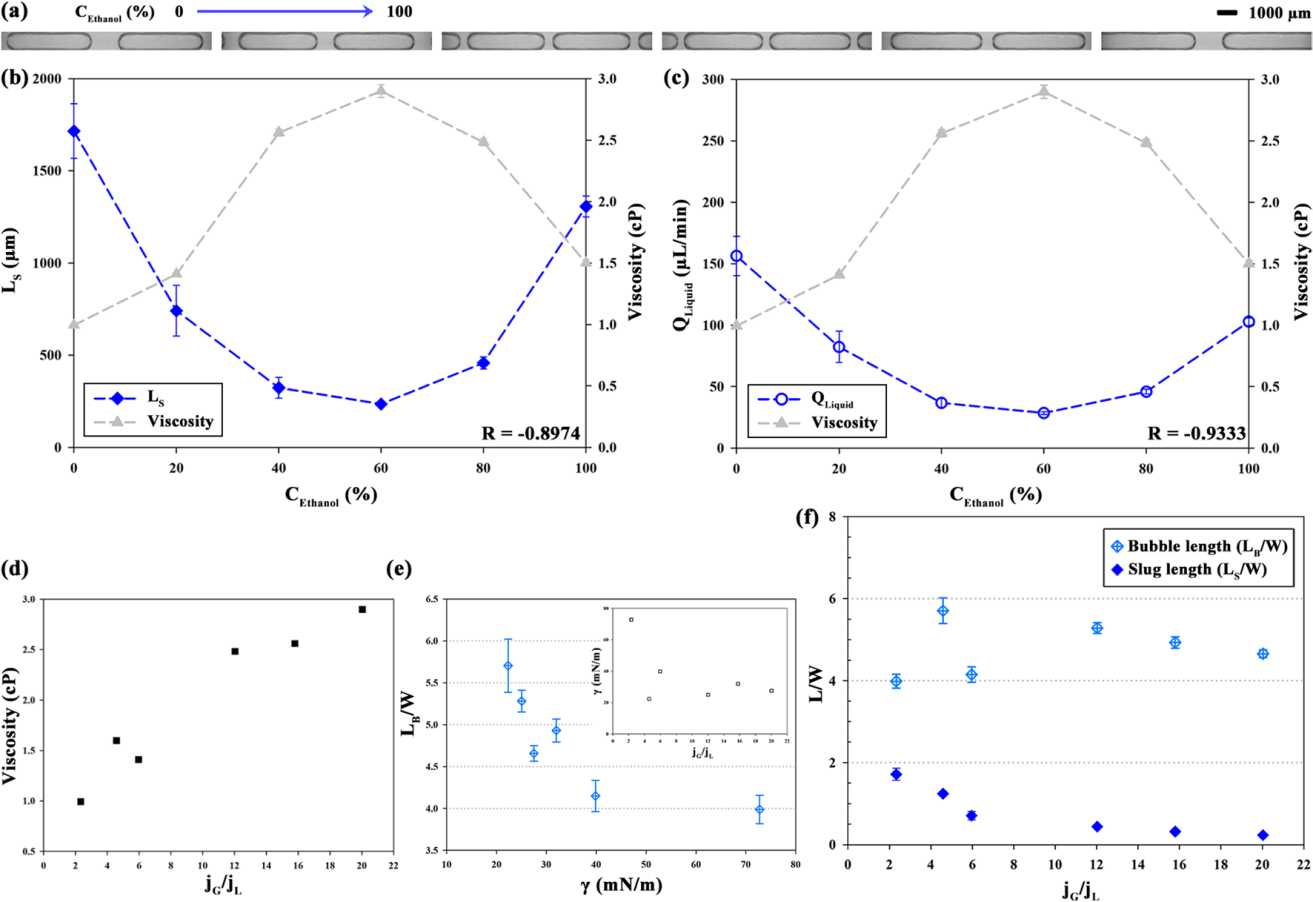
(**a**) Representative images in 3D-printed chip depending on the volume percent of ethanol [*C*_Ethanol_ (%)] for ethanol–water mixture. (**b**) Length (*L*_S_) and (**c**) flow rate (*Q*_Liquid_) of slugs depending on the viscosity of liquid depending on *C*_Ethanol_. Viscosity of ethanol–water mixture was overlapped depending on *C*_Ethanol_ (%). Correlation coefficients for the viscosity of liquid are R = –0.8974 (*L*_S_) and R = –0.9333 (*Q*_Liquid_). (**d**) Air–liquid superficial velocity ratio (*j*_G_/*j*_L_) depending on viscosity. (**e**) Length of bubble (*L*_B_/W) with surface tension (γ) and the plot of surface tension (γ) with *j*_G_/*j*_L_ in the inset. (**f**) Dimensionless length (*L*_S_/W, *L*_B_/W) versus *j*_G_/*j*_L_.

The resistance of liquid part (*R*_Liquid_) is changed by varying the viscosity of the liquid sample used as the working fluid. In this study, *R*_Liquid_ is proportional to the viscosity of the mixture of ethanol and water, as expressed in Eq. (4). The viscosity of the mixture varies with ethanol concentration (volume percent of ethanol; *C*_Ethanol_). The *Q*_Pump_ is fixed at 500 µL/min and *C*_Ethanol_ is varied from 0 to 100%. Figure 5a–c illustrates *L*_S_ and *Q*_Liquid_ depending on *C*_Ethanol_, and the overlapped viscosity (Pa s/mm^3^) of ethanol–water mixture, which was measured under the same condition of *C*_Ethanol_. The viscosity of mixture has the highest value at 60% of C_Ethanol_. Based on the viscosity, the minimum and maximum values of *R*_Liquid_ are 0.32 and 0.94 Pa s/mm^3^, respectively, at relevant *C*_Ethanol_. The increase in *R*_Liquid_ results in the decrease in the flow rate of the liquid (*Q*_Liquid_) at constant Δ*P* (Δ*P* = *R*_Liquid_*Q*_Liquid_). The value of *L*_S_ is the minimum (*L*_*S*_ = 233 µm) at *C*_Ethanol_ = 60%, and the maximum (*L*_*S*_ = 1716 µm) for water, i.e., at *C*_Ethanol_ = 0%. The values of *Q*_Liquid_ also show a similar trend with a minimum value (*Q*_Liquid_ = 28 µL/min) at *C*_Ethanol_ = 60% and a maximum value (*Q*_Liquid_ = 156 µL/min) at *C*_Ethanol_ = 0%. Both parameters have the minimum value at 60% of C_Ethanol_, which is in accordance with the maximum viscosity of the working fluid. The correlation coefficient between the viscosity of ethanol–water mixture and *Q*_Liquid_ is R = − 0.9333, and between *L*_S_ and *Q*_Liquid_ is R^2^ = 0.9705.

For detailed analysis, Fig. 5d–f represent the variation of *j*_G_/*j*_L_ and normalized length of slug and bubble. Figure 5d shows the change of *j*_G_/*j*_L_ with viscosity of mixture because viscosity is related with *j*_G_/*j*_L_ linearly in general since *Q*_Liquid_ decreases with increased *R*_Liquid_ (Eqs. (1) and (4)). In Fig. 5e, the relation within *L*_B_/W, *j*_G_/*j*_L_, and surface tension (γ) was depicted since γ has relevance to the force balance for generating the segmented flow. *L*_B_/W decreases with increased γ, and the inset shows the surface tension is inversely proportional to *j*_G_/*j*_L_. From that, high value of *j*_G_/*j*_L_ is under the conditions of relatively high viscosity and low γ while low *j*_G_/*j*_L_ is under the opposite. In Fig. 5f, *L*_S_/W is decreased with *j*_G_/*j*_L_ since the *R*_Liquid_ is increased by viscosity of liquid. By comparison, *L*_B_/W is remarkably affected by the variation of surface tension and density, especially in the lower *j*_G_/*j*_L_ range (*j*_G_/*j*_L_ = 2.33 ~ 5.96). In this part, high *j*_G_/*j*_L_ with low *L*_B_/W means that air occupies large proportion with small-size air bubble at high generation rate.

### Pump flow rate

**Figure 6:**
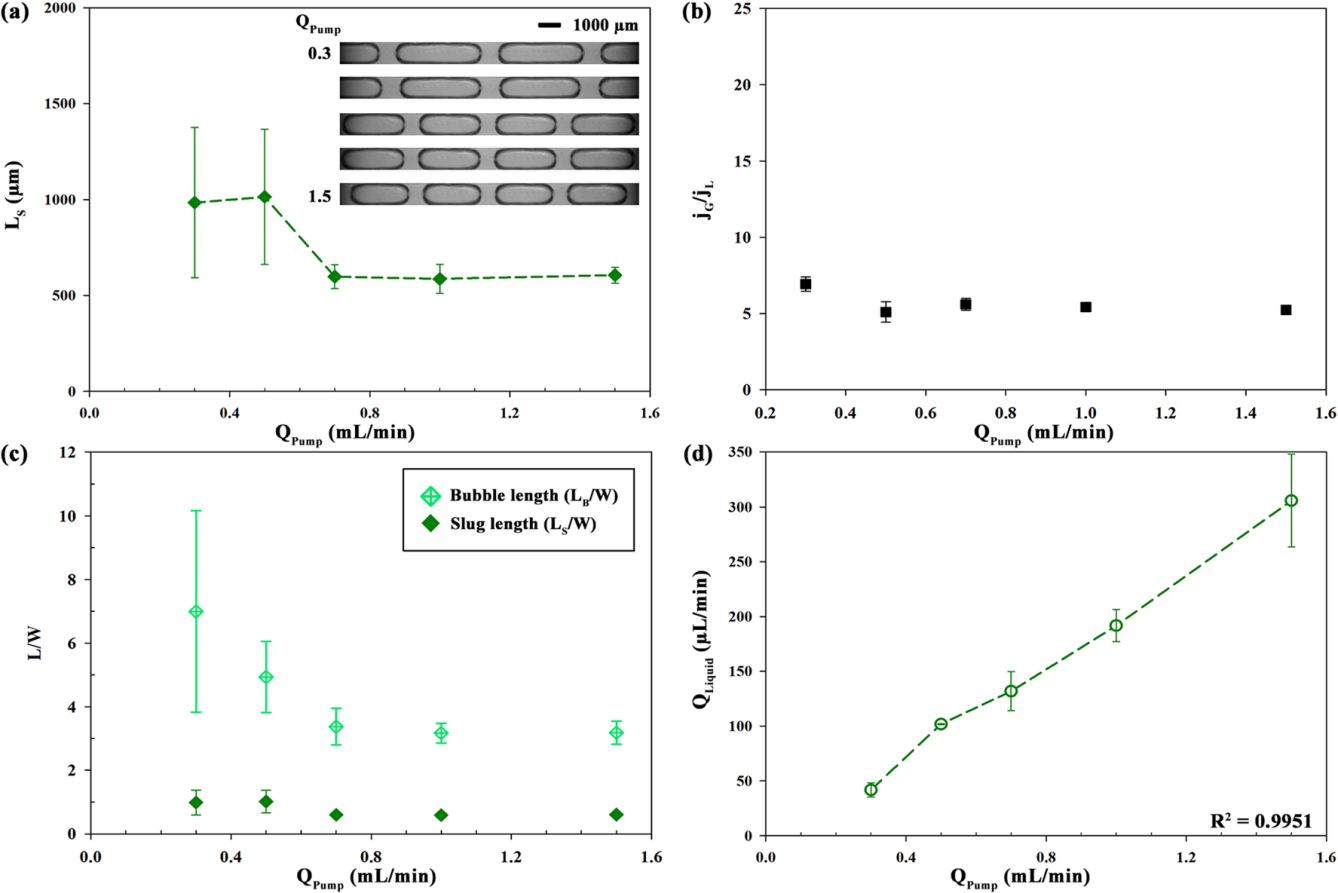
(**a**) Length of slugs (*L*_S_) depending on pump flow rate [*Q*_Pump_ (mL/min)] of the syringe pump and representative images in 3D-printed chip as an inset of plot. (**b**) Air–liquid superficial velocity ratio (*j*_G_/*j*_L_), (**c**) dimensionless length (*L*_S_/W, *L*_B_/W) and (**d**) flow rate of slugs (*Q*_Liquid_) depending on *Q*_Pump_. Correlation coefficients between *Q*_Liquid_ and *Q*_Pump_ is R^2^ = 0.9951 (*Q*_Liquid_).

To investigate the effect of negative pressure on the segmented flow, the *Q*_Pump_ of the syringe pump was controlled. From Eq. (1), Δ*P* increases with increased *Q*_Pump_ when *R*_*h*_ is constant. The length of the tube for *R*_Air_ (76 Pa s/mm^3^) was 400 mm and *C*_Ethanol_ for *R*_Liquid_ (0.32 Pa s/mm^3^) was 100%. The values of *Q*_Pump_ were changed as 300, 500, 700, 1000, and 1500 µL/min. In Fig. 6a, for the case of low *Q*_Pump_ (300, 500 µL/min), relatively large value of *L*_S_ is observed because it is difficult for the air flow to penetrate the liquid flow in the main channel due to the surface tension force (Fig. 2a). As *Q*_Pump_ increases from 500 to 700 µL/min, the time required for the growth of air bubbles decreases and there is an unanticipated decrease of *L*_S_ to 597 µm. For Fig. 6b, *j*_G_/*j*_L_ is not affected by *Q*_Pump_ since the ratio of *R*_Air_ and *R*_Liquid_ is nearly constant. In addition, *L*/W in Fig. 6c shows relatively high values with high deviations at *Q*_Pump_ = 300, 500 µL/min. It is related with the balanced forces such as interfacial tension, shear stress, and hydrostatic pressure. However, while the trends in *L*_S_/W are irregular with the increase in *Q*_Pump_, *Q*_Liquid_ shows an increasing trend in the range of 42–306 µL/min because *Q*_Pump_ regulates the total volume flow rate (*R*^2^ = 0.9951). This indicates that the time required to squeeze the bubbles gradually decreases by increasing *Q*_Pump_ from 700, 1000 to 1500 µL/min, despite similar sizes of liquid slugs (*L*_S_ = 597, 586, and 605 µm).

### Validation for squeezing regime

**Figure 7:**
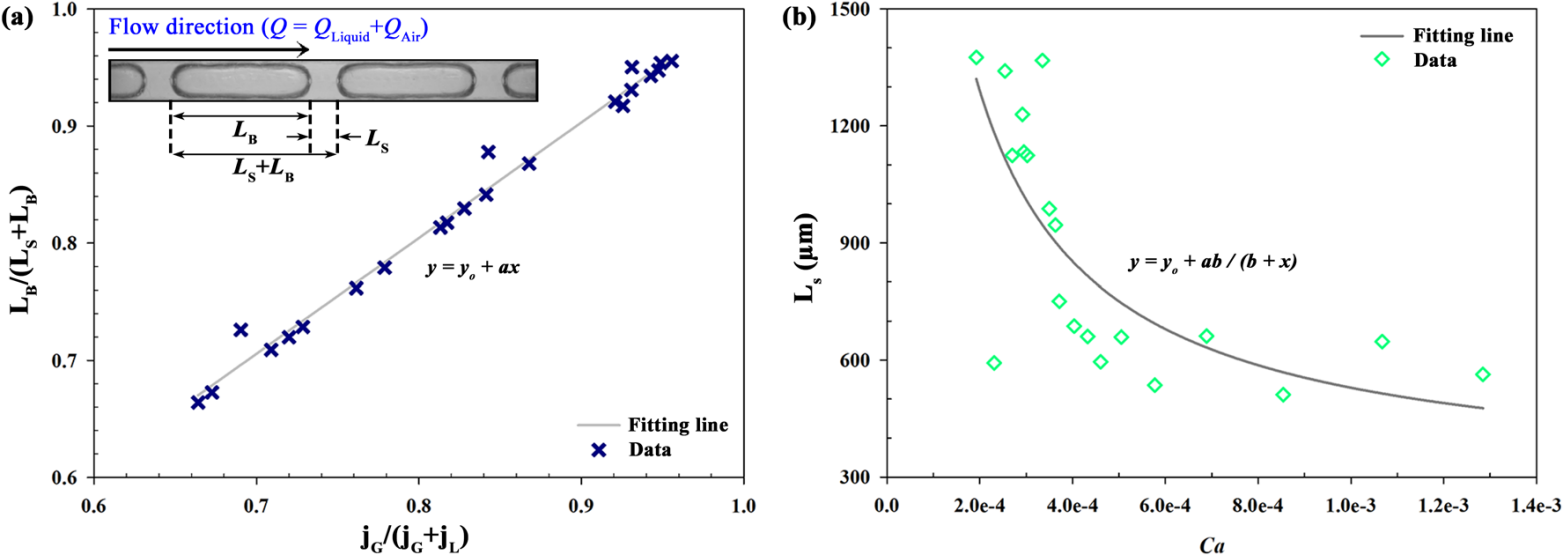
(**a**) Void fraction (*L*_B_/(*L*_S_ + *L*_B_)) depending on gas volumetric quality (*j*_G_/(*j*_G_ + *j*_L_)) (y_0_ = 0.0153, a = 0.9850). (**b**) The length of liquid slug (*L*_S_) with capillary number (*Ca*) (y_0_ = 280.6407, a = 4295.0156, b = 6.1407 × 10^–5^).

To validate the results under various conditions, Fig. 7a compares the values between void fraction (*L*_B_/(*L*_S_ + *L*_B_)) and volumetric quality (*j*_G_/(*j*_G_ + *j*_L_)). *L*_B_/(*L*_S_ + *L*_B_) is proportion of the bubble over the sum of liquid slug and air bubble (Fig. 3). *j*_G_/(*j*_G_ + *j*_L_) is proportion of gas flow rate to total flow rate (*Q* = *Q*_Liquid_ + *Q*_Air_)^20,52–54^. The trend line was expressed by *y* = *y*_*0*_ + *ax* with *y*_*0*_ = 0.0153 and *a* = 0.9850. It correlates well with linear relationship as Armand-type^20,53,55^. Generally, *L*_B_/(*L*_S_ + *L*_B_) correlates with *j*_G_/(*j*_G_ + *j*_L_). Figure 7b shows the *L*_S_ with capillary number (*Ca*) from 10^–4^ to 10^–3^. *Ca* was calculated by using the following equation (Eq. 5). *Ca* indicating the ration between viscous drag forces and surface tension forces can be expressed as follow:

5 $$Ca= \mu u/\gamma$$

where *µ* is the viscosity, *u* is the speed of carrier fluid, and *γ* is the surface tension. *Ca* has a relation with *j*_G_/*j*_L_ while certain value deviates from the line due to effects of their viscosity and surface tension^45,46^. *L*_S_ is decreased with increasing *Ca* since the capillary number intensifies shearing effect of two fluids^56^. Higher *Ca* means increased viscous force and the interface between two phases experiences higher shear stress. Therefore, the air could easily penetrate the continuous liquid phase at the junction and then it results in the faster breakup.

### Mixing by segmentation

**Figure 8:**
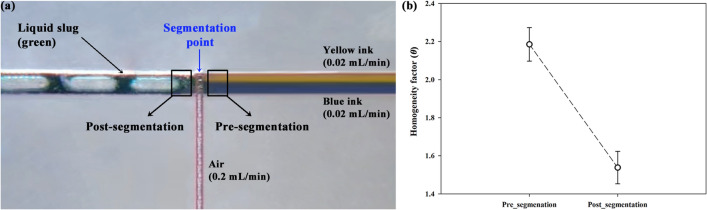
(**a**) Segmented flow in 3D-printed mixing chip (**b**) Homogeneity factor (*θ*) at pre- and post-segmentation.

The segmentation itself could influence on the mixing since molecular diffusion is generated by squeezing, stretching of liquid^14^. Figure 8a shows captured image of segmented liquid flow by air when two different inks (yellow and blue) enter the channel at 0.02 mL/min, respectively. Based on the segmentation point, the yellow and blue colors are mixed and then the segmented liquid slug has green color. According to Kašpar et al., The squeezing and stretching of liquid develop symmetrical circulation before the segmentation is finished^14^. Therefore, the homogenization is contributed by the molecular diffusion influencing the mixing efficiency. The homogeneity factor (*θ*) is expressed as following Eq. (6)^57^.

6 $$\theta = \sqrt{\frac{1}{M}{\sum }_{n=1}^{M}{({I}_{n}-\overline{I })}^{2}}/\overline{I }$$

where *M* is the number of pixels of liquid slug in the image, *I*_*n*_ is the intensity of each pixel point in the image, and *Ī* is the mean value of intensity distribution ($\overline{I }=\frac{1}{M}{\sum }_{n=1}^{M}{I}_{n}$). The value of *θ* decreases as the mixing is processed increasingly. In other words, the lower *θ* means the higher degree of mixing, and then the 0 means theoretically perfect mixing. However, in general, experiment and simulation analysis dealing with mixing efficiency have postulated that complete mixing is below 0.1 which is 90% degree of mixing. Figure 8b indicates the *θ* value at pre-segmentation and post-segmentation. Segmentation effect on mixing phenomenon was focused rather than other mixing mechanisms in this section. Therefore, data was extracted immediately before and after the segmentation. For accurate measurement, the image was acquired under the steady flow, and data was averaged by using 5 consecutive images. In addition, *θ* is calculated by including every intensity value per pixel in the ROI of 50 × 50 pixels. The decrease of *θ* means the progressed mixing state from the segmentation point as *θ* was decreased from 2.19 ± 0.09 to 1.54 ± 0.09. The degree of mixing is about 30% based on the normalized *θ*. The mixing performance by segmentation itself might differ depending on several parameters such as force balance and channel size.

## Discussion

The capillary numbers (*Ca*) were calculated under experimental conditions such as flow rate and fluid viscosity. In this study, the range of *Ca* is small (10^–4^ ~ 10^–3^). Given that the critical value distinguishing break-up regime of squeezing and shearing is about 10^–2^_,_ the squeezing break-up is observed in our 3D-printed chip^20^.

Figure 2 shows the squeezing regime at smaller *Ca*^58^. The carrier fluid (blue region) and the dispersed fluid (white region) form an interface, and the penetrating dispersed fluid grows steadily (Fig. 2a). As the air is injected in the T-junction of 3D-printed chip, the thread is distorted downstream (Fig. 2b). In this condition, there are three types of forces acting on the air bubbles for growing and squeezing: surface tension, shear stress, and pressure drop^16^. The Laplace pressure (Δ*P*_*L*_) related to the surface tension acts on the growing air bubbles as a stabilizing force, expressed as follows:

7 $${\Delta P}_{L}= \gamma \left(\frac{1}{{r}_{x}}+\frac{1}{{r}_{y}}\right)\approx \frac{2\gamma }{r}$$

where *γ* is the surface tension, *r*_*x*_ and *r*_*y*_ are the radii of curvature of each axis. From Eq. (7), Δ*P*_*L*_ can be simplified as 2*γ*/*r* because *r*_*x*_ and *r*_*y*_ are considered almost the representative radius (*r*) which is combination of *r*_*x*_ and *r*_*y*_. Forces induced by shear stress and pressure drop contribute to break-up of air–liquid interface. While the radius of the dispersed fluid (*r*) increases due to intake of the air, Laplace pressure of the interface is reduced. Subsequently, the collapse of neck produces the segmented flow within two immiscible fluids (Fig. 2c).

On the other hand, as the *Ca* (or flow rate) increases, the break-up regime is shifted to shearing regime such as dripping or jetting. In this regime, the dispersed fluid is broken up before it reaches the states shown in Fig. 2b^58^. When these forces were balanced under specific conditions, the dispersed fluid was squeezed at regular intervals. Thus, the uniform volumes of liquid slugs and bubbles were generated at a constant time interval^59^. When the air blocks the liquid flow and the pressure between the fluids reaches the balance (Fig. 2b), it indicates the end of the growth of bubbles^60^.

In Fig. 5, viscosity of liquid was changed by ethanol concentration (*C*_Ethanol_) and the surface tension was also varied with that. In a low *Ca*, the break-up regime is dominated by pressure drop (*ΔP*) so the length of air bubble and liquid slug (*L*_S_/W, *L*_B_/W) is generally determined by the ratio of the flow rate. However, the variation of *L*_B_ seems not to be proportional to air–liquid superficial velocity ratio (*j*_G_/*j*_L_) in Fig. 5f. It should be discussed in more detail. In this case, both viscosity and surface tension of the liquid part are different depending on the *C*_Ethanol_. In terms of surface tension effect on bubble generation, according to Garstecki et al., the balance of surface tension, static pressure and shear rate determines the *L*_B_^16^. Hao et al. also has reported the development of bubble in terms of relevant forces^61^. In T-junction channel, when gas penetrates liquid, destabilizing force is weak because static pressure and shear rate act in the other direction (Fig. 2a). However, when gas blocks the main channel (Fig. 2b), static pressure and shear rate are applied in the same direction (downstream) and then the neck of gas is pressed by the net force (Fig. 2c). The range of *∆P*_*L*_ (Eq. 7) is wider with the changing radius of curvature for high value of surface tension than low surface tension. Therefore, *∆P*_*L*_ of gas with high surface tension is considerably decreased when the gas blocks the main channel. At the same time, intensified destabilizing forces (shear stress (τ) and hydrostatic pressure (∆*P*) in Fig. 2b), which directs towards same direction, push the neck of gas downstream, and generate the gas bubble more easily. In fact, the surface tension has an impact on the attachment force and interface of the bubble during the bubble growth^62–64^. As a result, it can explain the relation between *L*_B_ and surface tension in Fig. 5e. In addition, in Fig. 5d, viscosity of liquid has an impact on the *j*_G_/*j*_L_ since flow rate of liquid (*Q*_Liquid_) decreases with increased resistance of liquid part (*R*_Liquid_) in Eq. (1). The increased viscous force acts on the interface between air and liquid, and the higher shear stress makes the air penetrate easily. In summary, viscosity, surface tension and flow rate intricately effects on the generation of *L*_B_. In other words, although *L*_B_ should increase with increased *j*_G_/*j*_L_ in Fig. 5f, the expected trend is weak under the condition of simultaneously changing surface tension contributing to decreased *L*_B_.

As shown in Fig. 6, under the low pump flow rate (*Q*_Pump_) condition, initially the surface tension force is dominant because the radius of the air tip (*r* in Eq. (7)) increases slowly. This means that Δ*P*_*L*_ is relatively large at an early stage in Fig. 2a,c, and thus, the stabilizing force is dominant over shear stress and pressure drop. Therefore, air–liquid flow takes time to segment each other due to the low flow rate and then *L*_S_/W and *L*_B_/W are longer in the case of low *Q*_Pump_ condition (300–500 µL/min). As destabilizing forces, there exist the shear stress force (*τ*) that is related to the flow rate of the liquid, and the resistance force that is related to the pressure drop (ΔP) over the bubbles based on the Hagen-Poiseuille equation. For *Q*_Pump_ above 700 µL/min in this experiment, the radius of the air tip (*r*) increased and Δ*P*_*L*_ decreased rapidly. Therefore, thread grew and blocked the main channel rapidly so that *L*/W decreased. Under the relevant condition (*Q*_Pump_ = 700 ~ 1500 µL/min), the air bubble was squeezed by the liquid stream immediately after the thread had grown up to a specific radius. Moreover, *j*_G_/*j*_L_ maintained a similar value with an average value of 5.4185 and standard deviation of 0.2650. This means that *L*_S_/W and *L*_B_/W have approximately the same value at *Q*_Pump_ = 700 ~ 1500 µL/min, whereas the flow rate of liquid slug (*Q*_Liquid_) increases under this *Q*_Pump_ condition. Consequently, as the total flow rate increases, the bubbles form with a higher frequency through the squeezing process forced by the shear stress and resistance. In other words, the pressure-driven flow by controlling *Q*_Pump_ determines the flow rate of each fluid through the flow channel, and allows liquid slugs to dynamically be changed such as length or frequency^65^. Further, the air bubble, which can be considered as a gap between neighboring slugs, also varies with different vacuum pressure.

For Fig. 8, influence of segmentation on the mixing was investigated based on the homogeneity factor. Wang et al. investigated the mixing performance in segmented liquid with respect to the channel width^66^. As the size of segmented liquid was reduced, the required distance for effective mixing was also reduced. In addition, Filatov et al. reported the increased viscosity generates vortex flow in liquid, and increases mixing index due to the proportional relation with shear stress^67^. According to the analysis of liquid viscosity with liquid slug (Fig. 5), increased *R*_Liquid_ with increased viscosity results in the decreased flow rate of the liquid (*Q*_Liquid_). Therefore, *j*_G_/*j*_L_ increases with viscosity of liquid while *L*_S_/W decreases. As a result, viscosity of liquid changes the L_S_/W, and then could influence the mixing efficiency of segmented flow.

In this paper, the volume of liquid slug from the overall results is within the range of 0.23 µL (*L*_S_ = 233 µm) to 1.72 µL (*L*_S_ = 1716 µm). It indicates the liquid flow segmented by the air could deal with the micro-scale samples.

## Conclusion

A vacuum-driven segmented air–liquid flow was created in a 3D-printed chip using a single-pump system. The size and flow rate of the liquid slugs were closely analyzed considering several parameters such as tube length of air flow, viscosity of liquid sample, and pump flow rate. In addition, the ratio of gas–liquid superficial velocity and air bubble size were also investigated in accordance with the force balance. Further, the degree of mixing was measured by homogeneity factor. Then, segmentation process itself is also considered as a factor influencing the mixing efficiency.

Both liquid and air flows can be controlled by modifying their resistances. In this study, the flow rate of the segmented liquid is directly proportional to the resistance, and the size of the liquid slug also shows a similar trend with the flow rate ratio. On the contrary, in case of air bubble for changing liquid properties to control liquid resistance, the portion occupied by the gas in the total flow gradually increases due to the increased viscosity, but the length of the bubble is considerably affected by the value of the surface tension. For air–liquid flow under the controlled total pressure, the size of the liquid slug shows abrupt reduction for the increased pump flow rate because several forces acting on the liquid slug and bubble interact.

These results indicate that the size and flow rate of the two-phase flow can be regulated by vacuum-driven flow using a single pump. Moreover, the understanding of the mechanism of slug or droplet formation can be enhanced in terms of pressure drop, flow rate, and resistance including liquid properties within the flow. However, there are still some areas that need to be considered as a follow-up study. One of the areas is the effect of surface property in the channel. It can affect the generation process of gas–liquid segmented flow^68^, and the configuration of liquid slugs can be altered by the channel surface such as wetting properties via coating^65^. 3D printing channel is also considered as research field in terms of influence of surface affinity on droplet generator^69^. Nonetheless, this study could contribute to the field of biosensor system such as sensitive diagnostic assays^5,70^, point-of-care testing (POCT), compact liquid-handling pump^71^, etc. Firstly, it can be applied to the field of diagnostic assays using chemical or biological droplet mixing since controlling the size of the liquid is related to the mixing efficiency. Secondly, it can be utilized for studies about portable biosensor such as POCT. Many related studies try to analyze and apply a system that forms a pressure-based flow for miniaturization of the pumping system. Ease-of-operation and portability make the potential for application higher through miniaturization using compact liquid-handling system. Therefore, the results in this study can contribute to improved access to various other pressure-driven flow-based studies since the control of two-phase fluid was conducted through various parameters based on the circuit model under vacuum-driven flow.
